# Before executive dysfunction is attributed to fibromyalgia, other causes must be thoroughly ruled out

**DOI:** 10.1055/s-0045-1805074

**Published:** 2025-05-26

**Authors:** Josef Finsterer

**Affiliations:** 1 Neurology & Neurophysiology Center, Vienna, Austria.

Dear Editor,

We were interested to read the article by Mendonca et al. on executive function in 17 women with fibromyalgia (FM) who were assessed using the Wechsler Abbreviated Scale of Intelligence (WASI), the Digit Span subtest of the Wechsler Adult Intelligence Scale (WAIS), the Five Digit Test (FDT), the Trail Making Test (TMT), the Corsi Block Tacking Test (CBTT), the Hayling Test (HT) and the Verbal Fluency Task (VFT). 1

It was found that FM women had prolonged response latency on the HT and TMT, made fewer errors on the B-HT, and performed worse on the DS backward as well as CBTT forward and backward. 1 There were moderate correlations in the expected direction between performance on the CBTT backward and interference at work, and between time to completion of the B-TMT and fatigue. 1 There was also a relationship between errors on the B-HT and interference at work. 1 It was concluded that FM women have reduced efficiency in inhibitory control and cognitive flexibility and have difficulties in working memory and nonexecutive processes such as processing speed. The study is convincing, but some points should be discussed.

The first point is that the causes of executive dysfunction other than FM were not sufficiently ruled out. To adequately rule out alternative causes, it would have been imperative to examine the 17 FM women neurologically by means of cerebral imaging, electroencephalography (EEG), and electromyography.

The second point is that test–retest reliability was not investigated. To assess whether the results are truly reliable, it would have been imperative to repeat the tests at a second time point.

The third point is that no control group was included in the study. Since neither health nor disease controls were included in the analysis, the results are even less reliable.

The fourth issue is that the diagnostic criteria for FM applied to the 17 women were not specified. The diagnosis of FM was made by a rheumatologist, but the criteria used were not mentioned. 2 Were the diagnostic criteria of the American Association of Rheumatologists applied?

The fifth point is that FM is also characterized by myalgia. 3 We should know whether the degree of myalgia was assessed and whether muscle pain correlated with any of the neuropsychological tests used.

The sixth point is that the current medication that the 17 included women were regularly taking was not included in the evaluation. In order to exclude the influence of their current medication on test performance, it would have been imperative to provide this information.

In summary, the index study has limitations that put the results and their interpretation into perspective. Addressing these limitations could strengthen the conclusions and support the results of the study. Before attributing cognitive dysfunction to FM in this population of female patients, all alternative etiologies must be thoroughly ruled out.

## References

[1] Mendonça B TV Machado V Silva G G Dias N M Executive functions and functioning in women with fibromyalgia Arq Neuropsiquiatr 2024 82 09 1 9 10.1055/s-0044-1790577 39317225

[2] Bhargava J Hurley J A Fibromyalgia[Updated 2023 Jun 11]. In: StatPearls [Internet].Treasure Island (FL) StatPearls Publishing 2024Jan-. Available from:https://www.ncbi.nlm.nih.gov/books/NBK540974/

[3] Jurado-Priego L N Cueto-Ureña C Ramírez-Expósito M J Martínez-Martos J M Fibromyalgia: A Review of the Pathophysiological Mechanisms and Multidisciplinary Treatment Strategies Biomedicines 2024 12 07 1543 10.3390/biomedicines12071543 39062116 PMC11275111

